# Nutrition knowledge, food choices and diet quality of genotyped and non-genotyped individuals during the COVID-19 pandemic

**DOI:** 10.1177/02601060211026834

**Published:** 2022-12

**Authors:** Angeliki Kapellou, Gabriela Silva, Leta Pilic, Yiannis Mavrommatis

**Affiliations:** 62693 St Mary’s University Twickenham, London, UK

**Keywords:** Nutrition knowledge, food choices, dietary intakes, diet quality, behaviour change, genotype-based advice, personalised nutrition, COVID-19

## Abstract

**Background:** Severe obesity (body mass index ≥ 40 kg/m^2^) and non-communicable diseases, both influenced by diet, have been associated with COVID-19. Genotype-based personalised nutrition advice may improve nutrition knowledge and enhance behaviour change towards better diet quality compared with conventional recommendations. **Aim:** To investigate the nutrition knowledge, food choices and diet quality in genotyped and non-genotyped individuals during the COVID-19 pandemic. **Methods:** One hundred and twenty-three healthy UK adults were recruited using convenience sampling through social networks. The online questionnaire consisted of the General Nutrition Knowledge Questionnaire, the Food Choices Questionnaire, and the EPIC-Norfolk Food Frequency Questionnaire (FFQ). FFQ was used to calculate participant diet quality with the Diet Quality Index-International and socio-demographic and anthropometric data. **Results:** Median general nutrition knowledge, diet variety and diet balance scores were higher in genotyped compared with non-genotyped individuals (71.0 ± 11.0 *vs*. 61.0 ± 15.0, *p* = <.001, 18.00 ± 5.00 *vs*. 15.00 ± 5.00, *p* = .007 and 2.00 ± 4.00 *vs*. 0.00 ± 2.00, *p* = .025, respectively). Pooled sample multiple regression showed that health motive positively influenced while familiarity motive negatively influenced diet quality index scores (*β* = .428, *t* = 4.822, *p* = <.001 and *β* = –.356, *t* = –4.021, *p* = .001, respectively). **Conclusions:** Nutrition knowledge and diet quality indices of balance and variety were higher among genotyped compared with non-genotyped individuals; overall diet quality was similar between groups. This may be due to pandemic-specific factors, such as altered motives of food choice and availability.

## Introduction

In December 2019, the first cases of COVID-19, an acute respiratory syndrome caused by the novel coronavirus 2 (SARS-CoV-2) were reported in Wuhan, China (Zabetakis et al., 2020). Since the early stages of the pandemic, severe obesity (body mass index (BMI) ≥ 40 kg/m^2^), hypertension, cardiovascular disease and diabetes have been associated with increased susceptibility to COVID-19 infection, symptom severity and increased mortality (Palaiodimos et al., 2020; Zhou et al. 2020). With immune capacity being the common denominator, chronic inflammation is implicated in the onset and progression of non-communicable diseases (NCDs), while systemic inflammation may, in turn, aggravate COVID-19 symptoms (Zabetakis et al., 2020).

Worldwide authorities have responded to this crisis with measures such as social distancing, wearing masks, thorough hand sanitising and staying at home. However, while lockdown measures are shown to contain the spread of the virus, they have substantially changed individuals’ lives and have caused psychological distress and loss of income (Huizar et al., 2020; Jayawardena and Misra, 2020; Naja and Hamadeh, 2020). Recent studies showed that motives of health, mood and weight control were more important for individuals during lockdown compared with the pre-lockdown period (Marty et al., 2020; Snuggs and McGregor, 2020). Such investigations are particularly important because a change in food choice motives may affect dietary intakes (Rangel, 2013; Steptoe et al., 1995).

Several dietary compounds have been associated with immunomodulatory properties, including vitamins (C, D and E), minerals (zinc, copper), fibre, and bioactives such as flavonoids and probiotics (Bhushan et al., 2021). Provided that inflammation is the common denominator of disease, a healthy diet may protect from both NCDs and viral infections (Zabetakis et al., 2020). Indeed, malnutrition (i.e. undernutrition and overnutrition) has been associated with a higher risk for COVID-19 infection, suggesting that too little or too much are equally problematic (Huiza et al., 2020).

Nevertheless, diet has not been on the forefront of public health messages to fight the current pandemic and there are no evidence-based dietary strategies to attenuate COVID-19 severity. Accordingly, the World Health Organization suggests any healthy diet that provides all nutrient reference nutrient intakes (RNIs) and prevents nutritional deficiencies to safeguard population health (Naja and Hamadeh, 2020; Palaiodimos et al., 2020; Zabetakis et al., 2020).

Contrary to these recommendations, reports have shown decreased consumption of fresh foods (Bracale and Vaccaro, 2020) and increased consumption of snacks and ‘junk’ food (i.e. calorie-dense sweet and savoury meals) during lockdown compared with the pre-lockdown period (Di Renzo et al., 2020; Sidor and Rzymski, 2020). Indeed, systematic reviews to date report a positive, yet weak, relationship between nutrition knowledge and diet, suggesting that public health actions to increase nutrition knowledge are likely insufficient for promoting behaviour change towards healthy eating (Spronk et al., 2014; Worsley, 2002). Indeed, validated behavioural theories are used to predict the effectiveness of such interventions, since factors such as subjective norms, attitudes and perceived behavioural control are significant determinants of one’s intention to engage in behaviour change (Horne et al., 2020c).

Meanwhile, personalised nutrition is an approach that combines individual phenotypic, genetic and lifestyle information to develop tailored nutrition advice (Ordovas et al., 2018). A recent systematic review showed that personalised nutrition improves dietary intakes in healthy adults when compared with conventional dietary advice and, thus, personalised nutrition interventions may be used in the future as a strategy to improve healthy eating among populations (Jinnette et al., 2020). Research has also proposed the potential of personalised nutrition in the prevention of NCDs, through predicting individuals’ variability in response to diet (Franzago et al., 2020).

Currently there is lack of research exploring the food choice motives and the diet quality in the UK during the pandemic in the context of gene-based personalised nutrition advice (i.e. nutrigenomics). Therefore, the present study aims to investigate the nutrition knowledge, the motives of food choice and the diet quality in a cohort of UK adults during the COVID-19 pandemic and to compare between individuals that have received gene-based personalised nutrition advice and those that have received conventional diet recommendations. It was hypothesised that individuals that have been genotyped for their risk of NCDs and have received genotype-based personalised nutrition advice would have greater nutrition knowledge, are more motivated towards healthier food choices and have a better diet quality compared with those who are not aware of their genetic risk of NCDs and have received conventional diet recommendations.

## Methods

### Participants

The survey was approved by St Mary’s University Ethics Committee and conducted in agreement with the Declaration of Helsinki (World Medical Association, 2013). A sample size calculation was conducted to identify the proportion of the population that has been genotyped. A sample size of 122 participants was calculated based on a significance level of 0.05, margin for random error +/– 4 and a standard deviation (SD) of 22.5. Recruitment was conveyed through convenience sampling using social networks and institutional emails and participants completed the questionnaire directly on Jisc online platform in full anonymity. Participants included predominantly healthy adults between 18 and 65 years old living in the UK that provided written informed consent and were excluded in the case that they: a) did not understand or write English, b) had changed permanent home address in the past six months, c) had tested positive for COVID-19 during the past month, d) followed any type of restrictive diet, and e) had any nutrition and/or dietetics background. Participants were asked whether they had been genotyped and, among those who had been genotyped, additional questions were included regarding the reason for genotyping, whether they were aware of their genetic risk of NCDs and whether they had received any kind of counselling for their genetic test results. All individuals who had received any type of genetic information (with or without genetic-based nutrition recommendations) were included in the analysis. Respondents who stated that are aware of their non-genetic risk of NCDs such as family history, were excluded from the analysis.

### Demographics

Demographic questions included gender, age and ethnic origin. Socioeconomic status (SES) questions included level of education, current employment, occupation, income and whether employment status and income had changed during the COVID-19 pandemic. A composite SES score was calculated by adding all SES components. Participant SES scores ranged between 6 and 24, with the highest qualification, income et cetera being scored with the lowest number and the lowest qualification, income et cetera with the highest number, thus the lower score indicating higher SES (Oakes and Rossi, 2003). Questions were also asked about weight, height, current diseases and dietary pattern followed.

### Nutrition knowledge

Nutrition knowledge was assessed using the validated revised version of the General Nutrition Knowledge (GNK) Questionnaire. The questionnaire includes 88 items regarding knowledge on expert dietary recommendations and the associations between diet and disease (Kliemann et al., 2016). To avoid response bias, participants were asked not to guess the correct answers and respond to the best of their knowledge. Participant GNK scores (GNKSs) were calculated by adding all items: for every correct answer, the score was 1 and for every wrong or ‘I don’t know’ answer, the score was 0, with a total GNKS ranging between 0 and 88.

### Food choices

Motives of food choices during the past month were assessed using the validated Food Choices Questionnaire. The questionnaire consists of 68 items regarding nine factors that influence food selection (health, mood, convenience, sensory appeal, natural content, price, weight control, familiarity, and ethical concern). The scores of food choices were calculated for each factor: a) ‘Not important at all’ = 1, b) ‘A little important’ = 2, c) ‘Moderately important’ = 3 and d) ‘Very important’ = 4. The average values per factor were used for analysis (Steptoe et al., 1995).

### Dietary assessment

The food intakes of participants during the past month were assessed using the validated EPIC-Norfolk Food Frequency Questionnaire (FFQ). The FETA software was used to calculate participant intakes (Mulligan et al., 2014) and the output was then used to calculate the Diet Quality Index-International (DQI-I) of individuals (Kim et al., 2003). This index uses a scoring from four components of diet quality: a) variety (0–20) (five food groups: fruits, vegetables, meat; poultry; fish; egg, dairy; beans, grains (0–15) and six sources of protein: dairy, eggs, meat, poultry, fish, beans (0–5)), b) adequacy (0–40) (eight groups: fruits, vegetables, fibre, grain, protein, vitamin C, iron, calcium), c) moderation (0–30) (five groups: sodium, empty calorie foods, total fat, saturated fat and cholesterol) and d) balance (0–10) (macronutrient and fatty acid ratio). Total diet quality index (DQI) was then calculated by compiling the individual scores, producing a number between 0 and 100. For further analyses, total DQI scores were dichotomised into ‘good’ and ‘poor’ categories, using the cut-off point of 60% of full DQI scores (Kim et al., 2003).

### Statistical analysis

Statistical analysis was performed using IBM SPSS Statistics 26.0. Data are shown as means ± SD or medians ± interquartile range for normally and non-normally distributed data, respectively. The differences in GNKS, food choices and DQI scores between genotyped and non-genotyped individuals were assessed using Independent samples *t*-test or Wilcoxon–Mann–Whitney *U* test, for normally and non-normally distributed data, respectively. Differences in categorical data between groups were assessed using the chi square test of independence or Fisher’s exact test, for expected cell counts > 5 and < 5, respectively. Association between total DQI scores and GNKSs of the total sample were evaluated using Spearman’s correlation coefficient. Multiple regression was used to explore the factors influencing total DQI within the total sample and adjusted for age, gender and SES score. Statistical significance was assumed at the 5% level.

## Results

### Participant characteristics

Main participant characteristics after excluding individuals who were aware of their non-genetic risk of NCDs (n=35), are shown in Table 1. In a predominantly healthy sample, five participants reported to have high or low blood pressure (4%), six reported to have high cholesterol (5%), while two reported to have diabetes (2%). While 71% (*n* = 87) of the sample did not follow a specific dietary pattern, the most-followed diet among respondents was the Mediterranean (*n* = 19, 15%). During the lockdown, 50% (*n* = 61) of the participants did not experience any change in their employment status, while 21% (*n* = 26) were in furlough and 29% (*n* = 36) worked from home. During the same period, 63% (*n* = 77) of the participants did not experience any change in their income, while in 31% (*n* = 38) of the participants their income decreased and in a small portion of them (6%, *n* = 8), it increased.

**Table 1.** Participant characteristics (*N*=123).

	**All** ***N*=123**	**Non-genotyped** ***n*=101**	**Genotyped** ***n*=22**	***p* value^c^**
**Males^a^**	36 (29)	26 (26)	10 (46)	.066
**Females^a^**	87 (71)	75 (74)	12 (54)	
**Age^b^ (years)**	31.0 ± 11.0	29.5 ± 10.0	35.0 ± 11.0	.071
**BMI^b^ (kg/m^2^)**	23.3 ± 5.5	23.2 ± 5.6	23.4 ± 4.6	.531
**SES score^b^**	13 ± 4	12 ± 4	14 ± 4	.050
**Ethnic group**			
**White^a^**	100 (81.3)	85 (84.2)	15 (68.2)	
**Asian^a^**	12 (9.7)	8 (7.9)	4 (18.2)	
**Latino/Hispanic^a^**	6 (4.9)	4 (3.9)	2 (9.1)	
**Mixed^a^**	4 (3.3)	3 (2.9)	1 (4.5)	
**Black^a^**	1 (0.8)	1 (1.1)	0 (0.0)	.259

^a^ Values represent *n* (%).

^b^ Values represent medians ± interquartile range.

^c^*p* value represents differences between genotyped and non-genotyped individuals.

BMI: body mass index; SES: socioeconomic status

Twenty-two of the respondents (18%) reported to have been genotyped, while 101 respondents had not (82%). There were no differences in the median age, BMI, SES score, or gender and ethnicity distribution between groups (Table 1). Most of the genotyped individuals were genotyped by 23andMe (*n* = 9, 41%), while the most popular reason for genotyping was diet (*n* = 16, 73%). Among the genotyped individuals, 54.5% (*n* = 12) had received an online report for their genetic results, 33.5% (*n* = 6) had received no report or counselling, while 12% (*n* = 4) had received an online or a face-to-face counselling session with a professional.

### Survey results

Participant scores on nutrition knowledge, food choice motives and diet quality are shown in Table 2.

**Table 2.** Participant scores on nutrition knowledge, food choices and diet quality.

	**All** ***N*=123**	**Non-genotyped** ***n*=101**	**Genotyped** ***n*=22**	***p* value^g^**
**GNKS (1–88)^a,b^**	65.5 ± 16.0	61.0 ± 15.0	71.0 ± 11.0	<.001
**Diet quality**				
**Good^c,^** ^d^	56 (48.3)	44 (46.3)	12 (57.2)	
**Poor^c,d^**	60 (51.7)	51 (53.7)	9 (42.8)	.369
**DQI, total (0–100)^e,f^**	59.09 ± 7.75	58.73 ± 7.66	60.71 ± 8.26	.290
**Variety (0–20)^e,b^**	15.00 ± 5.00	15.00 ± 5.00	18.00 ± 5.00	.007
**Adequacy (0–40)^e,f^**	27.81 ± 6.19	27.54 ± 5.89	29.05 ± 7.47	.314
**Moderation (0–30)^e,b^**	15.00 ± 6.00	18.00 ± 6.00	15.00 ± 11.00	.137
**Balance (0–10)^e,b^**	0.00 ± 2.00	0.00 ± 2.00	2.00 ± 4.00	.025
**Food choice scores (1–4)**				
**Health^b^**	3.00 ± 0.96	3.00 ± 0.83	3.67 ± 1.17	.003
**Mood^b^**	2.67 ± 1.00	2.50 ± 1.00	3.07 ± 1.09	.003
**Convenience^b^**	2.80 ± 1.00	2.80 ± 1.00	3.20 ± 1.00	.216
**Sensory appeal^b^**	3.25 ± 0.75	3.00 ± 0.75	3.50 ± 0.81	.010
**Natural ingredients^b^**	3.00 ± 1.00	3.00 ± 1.00	3.33 ± 1.00	.125
**Price^b^**	2.67 ± 1.00	2.67 ± 1.00	3.00 ± 1.42	.532
**Weight control^b^**	2.67 ± 1.33	2.67 ± 1.33	3.00 ± 1.09	.007
**Familiarity^b^**	1.75 ± 0.75	1.75 ± 0.75	2.13 ± 0.75	.006
**Ethical concern^b^**	2.00 ± 1.34	2.00 ± 1.34	2.00 ± 0.75	.544

^a^ GNKS.

^b^ Values represent median ± interquartile range.

^c^**’**Good**’** represents ≥ 60 and **‘**Poor**’** represents < 60 total DQI scores.

^d^ Values represent *n* (%).

^e^ DQI.

^f^ Values represent mean ± SD.

^g^*p* value represents differences between genotyped and non-genotyped individuals.

GNKS: General Nutrition Knowledge score; DQI: diet quality index

### Nutrition knowledge

The median GNKS of the whole sample was 65.5 ± 16.0. Distributions of the GNKSs significantly differed between groups, with genotyped individuals having significantly higher median GNKS than non-genotyped individuals (71.0 ± 11.0 *vs*. 61.0 ± 15.0, *p* < .001).

### Food choice motives

Motives of health, mood, sensory appeal, weight control and familiarity differed among groups, as shown by Mann–Whitney *U* test. Motives of convenience, natural ingredients, price and ethical concern were scored similarly between groups (*p* > .05) (Table 2).

Within the genotyped group, 81.8% (*n* = 18) reported to consider their genetic predisposition to NCDs when choosing food, while 18.2% (*n* = 4) reported not to consider it. The reported reasons for not considering genetic risk were ‘habit’ (*n* = 1) and ‘results do not take into account my lifestyle’ (*n* = 1).

### Diet quality

FFQ data were excluded from the DQI analysis if they reported an abnormally low (< 800 kcal for men, < 600 kcal for women) or high (> 5000 kcal for men, > 4000 kcal for women) energy intake (*n* = 7) (Kim et al., 2003). The mean DQI of the whole sample was 59.09 ± 7.75, with 51.7% of respondents (*n* = 60) having a poor diet quality and 48.3% of respondents (*n* = 56) having a good diet quality.

Total DQI scores were similar in both groups, as shown by independent samples *t*-test (*p* > .05). Regarding individual diet quality scores, genotyped individuals had significantly higher median diet variety and balance scores than non-genotyped individuals (18.00 ± 5.00 *vs*. 15.00 ± 5.00, *p* = .007 and 2.00 ± 4.00 *vs*. 0.00 ± 2.00, *p* = .025, respectively). Scores of diet adequacy and moderation were similar between groups (*p* > .05) (Table 2).

### Nutrition knowledge, food choice motives and diet quality

A Spearman’s rank-order correlation showed a statistically significant, weak positive correlation between GNKS and total DQI scores of the whole sample (*r*_S_ = .235, *p* = .011). Multiple regression analyses after controlling for age, gender and SES score were performed to investigate the significant contributors of the sample’s diet quality. Results showed that food choices of health and familiarity accounted for 20.6% of the variance observed in total DQI scores (adjusted *R*^2^ = .206, *F* = 6.962, *p* = <.001). Health motive positively influenced DQI (*β* = .428, *t* = 4.822, *p* = <.001), while familiarity motive negatively influenced DQI scores (*β* = –.356, *t* = –4.021, *p* = <.001).

## Discussion

The aim of the present study was to explore and compare the nutrition knowledge, food choices and diet quality between genotyped and non-genotyped individuals during the COVID-19 pandemic in the UK. The results indicate that, while nutrition knowledge and diet quality indicators of variety and balance were higher among genotyped compared with non-genotyped individuals, overall diet quality was similar between groups.

### Nutrition knowledge and diet quality

In view of the established association between diet and health, numerous public health initiatives in the past decades have been aiming at increasing knowledge around nutrition to improve dietary intakes at a population level (Spronk et al., 2014). The present study demonstrates that nutrition knowledge among genotyped individuals was significantly higher compared with non-genotyped individuals. This finding can be explained, at least in part, by the fact that genotyped individuals are usually health-conscious individuals, they might be more interested in expert recommendations and nutrition knowledge may have preceded genotyping (Floris et al., 2020; Ordovas et al., 2018). In support of this, the majority of genotyped individuals reported diet as the main reason for genotyping. Another possible explanation is the relevance of nutrition advice personalised to the individuals’ needs. Previous reports have suggested that lack of relevance of nutrition information, for example, delivering information on diet and blood cholesterol to younger individuals, does not enhance retention of information among those individuals because it is not relevant to them (Worsley, 2002). On the other hand, the study design does not allow conclusions as to whether genotyped individuals were more health-conscious to begin with or whether the intervention (genotype-based dietary advice) increased their nutrition knowledge. Therefore, the present findings highlight the need for more tailored dietary advice to improve nutrition knowledge.

Regarding diet quality, the present study demonstrates a positive weak correlation with nutrition knowledge, which is in line with previous research (Spronk et al., 2014; Worsley, 2002). Indeed, whereas nutrition knowledge is a central component of health literacy, other factors may influence the ability to interpret knowledge into a healthy diet, including an individual’s subjective norms and attitudes (Horne et al., 2020; Spronk et al., 2014; Steptoe et al., 1995). During the pandemic, a major factor that might have influenced this association would be the reduction in food availability in the market caused by the lockdown measures, such as decreased supply of fresh and imported products (Jayawardena and Misra, 2020; Naja and Hamadeh, 2020). As good diet quality improves cardio-metabolic and overall health, aiming at nutrition education strategies that would effectively improve individuals’ diet quality may protect them against severe illness due to infection (Naja and Hamadeh, 2020; Zabetakis et al., 2020).

### Risk of NCDs and diet quality

Considering the identified burden of NCDs on COVID-19 infection (Palaiodimos et al., 2020; Zhou et al., 2020), it was hypothesised that genotyped individuals with known genetic risk for NCDs would have a better diet quality compared with non-genotyped individuals. Despite the fact that genotype-based dietary advice may have contributed to higher scores of balance and variety, it did not appear to affect the diet in terms of adequacy, moderation and overall quality (Supplemental Figure S1 online). Although most genotyped individuals reported to consider their predisposition to NCDs when choosing food, it was not investigated whether these individuals were at high or low risk. In effect, studies on behaviour change based on provision of information on the apolipoprotein E (APOE) gene are conflicting, with some showing that knowledge of genetic risk enhances health behaviour change (Chao et al., 2008), while others did not show an effect (Fallaize et al., 2016), depending on the risk communicated. Indeed, it is plausible that individuals who do not carry the risk variant of the APOE gene for hypercholesterolaemia and Alzheimer’s disease were not motivated to change dietary behaviour.

Moreover, personalised nutrition advice has been shown more effective in improving diet compared with general population-based recommendations, provided that it is not based solely on genetic but also on phenotype and lifestyle information (Anderson et al., 2018; Nielsen and El-Sohemy, 2014). In the present sample, 54.5% of genotyped individuals had received online reports, which are usually based solely on genotype, thereby compromising the relevance and effectiveness of personalised nutrition advice to facilitate dietary changes (Celis-Morales et al., 2017). Nevertheless, genetic reports provide a detailed nutrition guide for health-conscious individuals who are interested in improving their diet (Floris et al., 2020). This might explain why the diets of genotyped individuals had a greater variety of foods from all food groups and protein sources in their diet and better balance in macronutrient and fatty acid intakes compared with non-genotyped individuals. If genetic testing was provided by registered dietitians with training in nutritional genomics to provide evidence-based advice, as suggested in a recent critical examination of legal and ethical considerations for nutrigenetic testing, it may have resulted in improved behaviour change and perhaps a greater diet quality in the genotyped group (Horne et al., 2020a, 2020b). Indeed, the Academy of Nutrition and Dietetics states that registered dietitians can be viewed as the only objective experts in utilising nutritional genomics to individualise care (Braakhuis et al., 2020).

### Food choice motives and diet quality

Multiple regression showed that food choices of health and familiarity accounted for 20.6% of the variance observed in total DQI scores. Health motive had a positive influence on DQI, indicating that health motive may be a facilitator of good diet quality, which is consistent with previous findings (Steptoe et al., 1995). On the other hand, familiarity had a negative influence on DQI, indicating that motive of familiarity may be a barrier of good diet quality. Familiarity tends to be associated with tradition and a healthy diet may not be adopted if advice deviates from the usual diets of individuals (Rankin et al., 2018). On the other hand, it has been previously shown that in those who are health driven, the least important factor when choosing food was familiarity (Marsola et al., 2020).

Regarding the motives of food choice between the groups, motives of health, weight control, mood, sensory appeal and familiarity were ranked higher among genotyped than non-genotyped individuals (Supplemental Figure S2). Indeed, individuals highly motivated to choose food based on health and weight control hold positive attitudes towards personalised nutrition (Rankin et al., 2018) and those motives are shown to be positively associated with diet quality during the lockdown (Marty et al., 2020).

Inconsistent with the present findings, previous research has shown that motives of mood and sensory appeal, which are negatively associated with diet quality, are not important among individuals that have adopted personalised nutrition advice (Rankin et al., 2018). Moreover, it has been shown that personalised nutrition advice may not be adopted if advice is different from the usual diets of individuals, especially if they are highly motivated by familiarity when choosing food (Rankin et al., 2018). Such inconsistencies may be due to changes during the pandemic which may have altered the food choice motives of individuals (Snuggs and McGregor, 2020). These motives being higher in genotyped individuals may also explain why diet quality was not higher in this group compared with non-genotyped individuals.

### Strengths and limitations

To the authors’ knowledge, this is the first study investigating individuals’ diet quality during the COVID-19 pandemic in the UK. The use of DQI-I in evaluating diet quality instead of single nutrients is a more holistic approach to explore diet, given that it integrates diet quality components such as adequacy rather than individual nutrient RNIs. Furthermore, the use of validated questionnaires to investigate nutrition knowledge, motives of food choice and dietary intakes of individuals provided robustness in study design.

A major limitation was that the nature of the study design precluded the ability to determine whether personalised nutrition motivated dietary change, or whether personalised nutrition consumers were more health-conscious and health literate before they received personalised nutrition advice. Moreover, the cross-sectional design of the study did not allow for comparisons between nutrition knowledge, food choice motives and diet quality pre- and post-pandemic and the data provided by participants were self-reported. In addition, large differences in sample sizes may have reduced the statistical power and therefore ability to detect actual significant differences between groups. Since eating behaviours and subsequent diet quality are often shared amongst household members, another limitation is that the present study did not consider household size (Fulkerson et al., 2014). Last, although participants were asked whether they are aware of their risk for NCDs, it was not investigated whether the risk was higher or lower. Since lower risk would not promote behaviour change towards better diet quality (Fallaize et al., 2016), this may have affected the results and future studies should ensure that this is taken into account.

## Conclusion

In a time of a global pandemic, balanced diets among individuals are a necessity. While nutrition knowledge and indicators of diet quality of balance and variety were higher among genotyped than non-genotyped individuals, overall diet quality was similar between groups, and this may be due to influences by pandemic-specific factors, such as altered motives of food choice and food availability. Interventions that would promote motives of healthy food choices to improve diet quality are warranted to effectively reduce the burden of NCDs and safeguard populations from this novel virus.

## Supplemental material

Supplemental Material, sj-docx-1-nah-10.1177_02601060211026834 - Nutrition knowledge, food choices and diet quality of genotyped and non-genotyped individuals during the COVID-19 pandemic Click here for additional data file. Supplemental Material, sj-docx-1-nah-10.1177_02601060211026834 for Nutrition knowledge, food choices and diet quality of genotyped and non-genotyped individuals during the COVID-19 pandemic by Angeliki Kapellou, Gabriela Silva, Leta Pilic and Yiannis Mavrommatis in Nutrition and Health

Supplemental Material, sj-docx-2-nah-10.1177_02601060211026834 - Nutrition knowledge, food choices and diet quality of genotyped and non-genotyped individuals during the COVID-19 pandemic Click here for additional data file. Supplemental Material, sj-docx-2-nah-10.1177_02601060211026834 for Nutrition knowledge, food choices and diet quality of genotyped and non-genotyped individuals during the COVID-19 pandemic by Angeliki Kapellou, Gabriela Silva, Leta Pilic and Yiannis Mavrommatis in Nutrition and Health

Supplemental Material, sj-jpg-1-nah-10.1177_02601060211026834 - Nutrition knowledge, food choices and diet quality of genotyped and non-genotyped individuals during the COVID-19 pandemic Click here for additional data file. Supplemental Material, sj-jpg-1-nah-10.1177_02601060211026834 for Nutrition knowledge, food choices and diet quality of genotyped and non-genotyped individuals during the COVID-19 pandemic by Angeliki Kapellou, Gabriela Silva, Leta Pilic and Yiannis Mavrommatis in Nutrition and Health

Supplemental Material, sj-jpg-2-nah-10.1177_02601060211026834 - Nutrition knowledge, food choices and diet quality of genotyped and non-genotyped individuals during the COVID-19 pandemic Click here for additional data file. Supplemental Material, sj-jpg-2-nah-10.1177_02601060211026834 for Nutrition knowledge, food choices and diet quality of genotyped and non-genotyped individuals during the COVID-19 pandemic by Angeliki Kapellou, Gabriela Silva, Leta Pilic and Yiannis Mavrommatis in Nutrition and Health
