# Supplementary material for: Nutrition knowledge, food choices and diet quality of genotyped and non-genotyped individuals during the COVID-19 pandemic
Source: Nutr Health. 2021 Jun 22;28(4):693–700. doi: 10.1177/02601060211026834 (PMC9716056; doi:10.1177/02601060211026834)
Supplement: Supplemental Material, sj-docx-2-nah-10.1177_02601060211026834 - Nutrition knowledge, food choices and diet quality of genotyped and non-genotyped individuals during the COVID-19 pandemic [file sj-docx-2-nah-10.1177_02601060211026834.docx]

**Titles and descriptions for Figures in Supplemental material**

**Figure S1.** Mean diet quality scores of total DQI, variety, adequacy, moderation, and balance in non-genotyped (n = 101) and genotyped (n = 22) individuals. * P < .05; Error bars represent ± SD.

**Figure S2.** Mean scores of food motives of health, mood, convenience, sensory appeal, natural ingredients, price, weight control, familiarity, and ethical concern in non-genotyped (n = 101) and genotyped (n = 22) individuals. * P < .05; Error bars represent ± SD.
