# Supplementary figures and images for: Nutrition knowledge, food choices and diet quality of genotyped and non-genotyped individuals during the COVID-19 pandemic
Source: Nutr Health. 2021 Jun 22;28(4):693–700. doi: 10.1177/02601060211026834 (PMC9716056; doi:10.1177/02601060211026834)

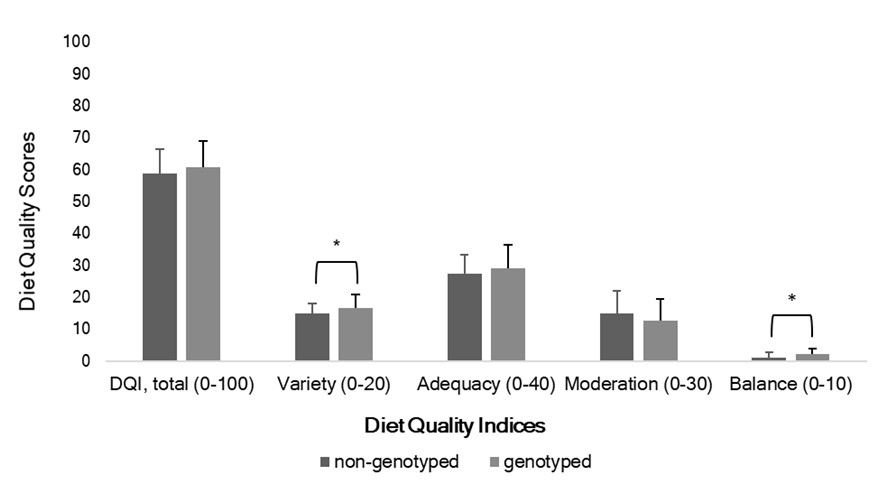

Supplement: Supplemental Material, sj-jpg-1-nah-10.1177_02601060211026834 - Nutrition knowledge, food choices and diet quality of genotyped and non-genotyped individuals during the COVID-19 pandemic [file sj-jpg-1-nah-10.1177_02601060211026834.jpg]

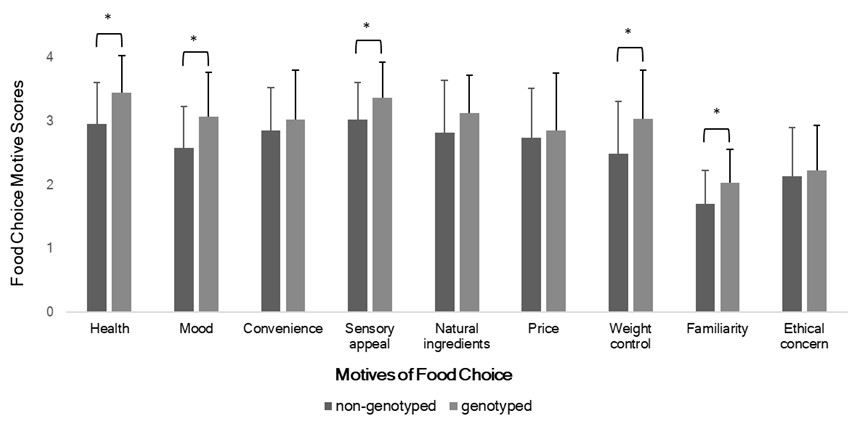

Supplement: Supplemental Material, sj-jpg-2-nah-10.1177_02601060211026834 - Nutrition knowledge, food choices and diet quality of genotyped and non-genotyped individuals during the COVID-19 pandemic [file sj-jpg-2-nah-10.1177_02601060211026834.jpg]
